# Polymorphisms in the glucocerebrosidase gene and pseudogene urge caution in clinical analysis of Gaucher disease allele c.1448T>C (L444P)

**DOI:** 10.1186/1471-2350-7-69

**Published:** 2006-08-03

**Authors:** Justin T Brown, Cora Lahey, Walairat Laosinchai-Wolf, Andrew G Hadd

**Affiliations:** 1 Asuragen, Inc., Austin, Texas, USA

## Abstract

**Background:**

Gaucher disease is a potentially severe lysosomal storage disorder caused by mutations in the human glucocerebrosidase gene (GBA). We have developed a multiplexed genetic assay for eight diseases prevalent in the Ashkenazi population: Tay-Sachs, Gaucher type I, Niemann-Pick types A and B, mucolipidosis type IV, familial dysautonomia, Canavan, Bloom syndrome, and Fanconi anemia type C. This assay includes an allelic determination for GBA allele c.1448T>C (L444P). The goal of this study was to clinically evaluate this assay.

**Methods:**

Biotinylated, multiplex PCR products were directly hybridized to capture probes immobilized on fluorescently addressed microspheres. After incubation with streptavidin-conjugated fluorophore, the reactions were analyzed by Luminex IS100. Clinical evaluations were conducted using de-identified patient DNA samples.

**Results:**

We evaluated a multiplexed suspension array assay that includes wild-type and mutant genetic determinations for Gaucher disease allele c.1448T>C. Two percent of samples reported to be wild-type by conventional methods were observed to be c.1448T>C heterozygous using our assay. Sequence analysis suggested that this phenomenon was due to co-amplification of the functional gene and a paralogous pseudogene (ΨGBA) due to a polymorphism in the primer-binding site of the latter. Primers for the amplification of this allele were then repositioned to span an upstream deletion in the pseudogene, yielding a much longer amplicon. Although it is widely reported that long amplicons negatively impact amplification or detection efficiency in recently adopted multiplex techniques, this assay design functioned properly and resolved the occurrence of false heterozygosity.

**Conclusion:**

Although previously available sequence information suggested GBA gene/pseudogene discrimination capabilities with a short amplified product, we identified common single-nucleotide polymorphisms in the pseudogene that required amplification of a larger region for effective discrimination.

## Background

Gaucher disease is a lysosomal storage disorder caused by mutations in the human glucocerebrosidase gene (GBA)[1] (for a review, see Grabowski[2]). There is considerable interest in clinical and research analysis of GBA. Assay methods have typically involved combinations of long-template PCR, gel electrophoresis, and southern blotting [3-5]. While these approaches are effective, there is a drive to take advantage of the improvements offered by multiplexed techniques[6]. A common analytical implementation of multiplexing involves the generation of multiple amplicons in a single PCR-based assay. Subsequent multiplex detection methods range from capillary electrophoresis to liquid-bead arrays [7,8] Importantly, it is well established that amplicon length bears heavily on amplification efficiency and that many recently adopted multiplex techniques display a preference for smaller amplicons [8-14] Multiplex assays are thus often developed from well-proven simplex PCR designs by reducing amplicon length. This and other technical changes present design challenges and can have unintended consequences that are not readily revealed in the controlled environment of the development laboratory.

For example, in order to design new human genetic assays with smaller amplicons, ample nucleotide sequence data sets are required. However, the sequence information required for quality design may not always be readily available, particularly in regard to the genomic diversity present in a given population. In the case of GBA, there are relatively few publicly available unique genomic DNA sequences [1,15,16] Sequences derived from cDNA are more abundant[17] but are not so useful as a design aid for assays targeting genomic DNA. Such design challenges in clinical molecular analysis of GBA are well established [5]. For example, a pseudogene (ΨGBA) exists downstream of GBA that is 96% identical to the functional gene [1]. Although ΨGBA is expressed[18], the presence of various defects, including a 55-bp deletion in exon 9, predict that it encodes a non-functional protein [1]. Interestingly, in all sequences reported to date ΨGBA also has an apparent defect paralogous to the 1448 T to C transition (c.1448T>C) that codes for a leucine to proline substitution at position 444 (L444P) in GBA [1,15,16]. When found in the functional gene, the c.1448T>C mutation can cause disease[19] and has been demonstrated in one system to reduce enzymatic activity by 77% [20]. In order to accurately perform genetic analysis of GBA, it is therefore imperative to distinguish the functional gene from the pseudogene. Unfortunately, the c.1448T>C transition falls in a region of very high identity between GBA and ΨGBA. Attempts to address this issue have involved assay systems amenable to large amplicons, such as restriction digest with gel electrophoresis, gene sequencing, or selective amplification using the upstream 55-bp deletion within ΨGBA. However, these designs are not predicted to be ideal for more recently developed techniques – such as real-time PCR and suspension bead arrays – that are being adopted by high-throughput clinical laboratories [3-5,8-14,21].

Using publicly available genomic DNA sequences [1,15,16], we developed multiplexed suspension bead array reagents to characterize alleles covering 8 diseases that are highly prevalent in the Ashkenazi Jewish population. The diseases assayed have a collective carrier frequency of 1:6 in this population: Tay-Sachs, Gaucher type I, Niemann-Pick types A and B, mucolipidosis type IV, familial dysautonomia, Canavan, Bloom syndrome, and Fanconi anemia type C. While assays for these diseases have generally involved multiple diagnostic platforms [3-5,22], the Signature Ashkenazi Carrier Panel (ACP) reagents allow for simultaneous genetic determinations of all diseases. This report details our identification of common ΨGBA and GBA polymorphisms not reported in the literature. These results demonstrate the importance of clinical evaluation and assay redesign in reducing the false positive frequency in a multiplex genetic test.

## Methods

### Reagents and samples

Solutions of 1M Tris-HCl, pH 8.0, 1X TE (10 mM Tris, 1 mM EDTA, pH 8.0), 10 % SDS, Signature Amp Mix I and nuclease-free water were obtained from Ambion Diagnostics (Austin, TX). Uracil-N-Glycosylase (HK-UNG) was obtained from EpiCentre (Madison, WI). AmpliTaq Gold and 10X Gold Buffer were obtained from Applied Biosystems (Foster City, CA). Deoxynucleotides were obtained from Bioline (Randolf, MA). Tetramethyl ammonium chloride (TMAC), sodium sarkosyl, and 2-(N-Morpholino)ethanesulfonic acid (MES) were purchased from Sigma (St. Louis, MO). The MES was adjusted to pH 4.5 by addition of 5 N NaOH then stored at 4C. Streptavidin-β-phycoerythrin (SA-PE) was obtained from ProZyme (Alameda, CA). EDC, (1-ethyl-3-[3-dimethylaminopropyl] carbodiimide hydrochloride), was obtained from Pierce (Rockford, IL). xMAP carboxylated microspheres were obtained from Luminex (Austin, TX). Human genomic DNA samples obtained from the Coriell Institute for Medical Research (Camden, NJ) were purchased as DNA isolated from cell lines. Oligonucleotides were synthesized by Biosearch Technologies (Novato, CA). All oligonucleotides were reverse-phase cartridge purified and prepared as 100 μM stock solutions in deionized water. Residual patient genomic DNA samples were used in this study. Samples were randomly selected and provided without coding or identifying information to members of the research team. Investigators were not provided with previously determined genotypes until after testing was completed. In addition, the investigators did not have access to the laboratory information systems of the facilities.

### PCR amplification

PCR reactions were prepared in 50-μL volumes using 5 mM Mg^2+^, 5 U of AmpliTaq Gold, 0.1 U of HK-UNG, and amplification primers. Either 0.8 pmol of each primer GDL444P-US1 and GDL444P-DS1 or, after clinical evaluations and redesign, 1.6 pmol of each primer GDL444P-U6 and GDL444P-DS1 was included. PCR primers for array detection and primers for sequencing are listed in Table 1. The final nucleotide mixture was 0.2 mM dATP, dCTP, and dGTP, 0.05 mM dTTP, and 0.4 mM dUTP. Reactions consisted of incubation of UNG at 37C for 15 minutes, then activation of AmpliTaq Gold enzyme for 10 minutes at 95C followed by amplification over 35 cycles of 94C for 30 s, 61C for 90 s, and 72C for 90 s, followed by 72C for 2 minutes. Single-tube multiplex reactions were performed with primers to amplify alleles in the Signature ACP assay (data not shown). Sample identities were irreversibly blinded to laboratory members. These de-identified patient DNA samples were prepared via either Qiagen BioRobot 9604 or Genovision automated methods according to manufacturer directions. A total of 5 uL of patient DNA sample was used per reaction. DNA samples were not quantified.

**Table 1:** Oligonucleotides used in this work

Primer Name	5' to 3' Sequence
GDL444P-DS1	Biotin-TTTAGCACGACCACAACAGC
GDL444P-US1	CATCTGTTCCCACATTCAGC
GDL444P-U6	GGTGCGTAACTTTGTCGACAGTCC
GDLwt	Aminododecyl-AGAACGACCTGGACGCAG
GDLmut	Aminododecyl-AGAACGACCCGGACGCAG
GDLseq-L1	AAGCTGAGAGTGTGATCCTGCCAA
GDLseq-L2	CCACAGCAGGATCCTTGATGGTAA
GDLseq-U4	GGACCGACTGGAACCCATCA

### Capture probe coupling

Oligonucleotide capture probes were modified at the 5' terminus with an aminododecyl group to allow conjugation to the carboxylated microspheres. The probes used are listed in Table 1. Covalent attachment of the amine modified probe to the carboxyl groups on the surface of the fluorescently addressed microspheres were completed using a standard carbodiimide coupling procedure [23]. Briefly, 5 × 10^6 ^microspheres were pelleted, resuspended in 50 μL of 100 mM MES, pH 4.5, then mixed with 200 pmol of aminododecyl-modified oligonucleotide. A 2.5 μL aliquot of 10 mg/mL EDC was added, the solution briefly vortexed and allowed to incubate for 5 minutes. A fresh 10 mg/mL solution of EDC was prepared for a second addition of EDC and 5 minute incubation. The microspheres were then washed with 0.02% Tween-20, then with 0.1% SDS, and then resuspended in 100 μL of 1X TE buffer, pH 8.0. Coupled microspheres were stored at 4C in the dark.

### Bead-array hybridization

A multiplex bead array containing c.1448T, c.1448T>C, and 43 additional capture probes was prepared in 1X hybridization buffer (3.0 M TMAC, 50 mM Tris-HCl, 4 mM EDTA, and 0.1% Sarkosyl, pH 8.0) such that each of the 45 beads was at a final concentration of 100 beads/uL. Samples were analyzed by mixing 48 μL of the bead solution with a 2 μL portion of amplified products. The samples were hybridized at 95C for 5 minutes and 52C for 25 minutes. Following hybridization, the samples were immediately transferred to a heat block pre-equilibrated to 52C on the XYP stage of a Luminex 100 System. This was followed by the addition of 25 μL of a 40 ng/μL SA-PE solution that had been freshly diluted in 0.75X hybridization buffer (2.25 M TMAC, 37.5 mM Tris-HCl, 3 mM EDTA, and 0.075% Sarkosyl, pH 8.0). Median fluorescence intensity (MFI) values were obtained from a minimum of 100 beads. Allele ratios were calculated as the fraction of fluorescent signal of the test allele divided by the sum of all fluorescent signals in the allele group. For the c.1448T>C mutation, this could be expressed as: MFI_c.1448T>C_/(MFI_c.1448T>C _+ MFI_C.1448T_). Allele ratios and genetic determinations were confirmed by using the configurable software tool Signature Script version 2.0 (Asuragen, Austin, TX). This software package can address ethical and policy considerations by revealing only those alleles and/or diseases which have been requested by the physician or genetic counselor. This software package also allows configuration of analysis parameters, stores test data in a database, queries the database with or without sample identifiers, and produces sample and batch reports.

### DNA sequencing

The c.1448T>C regions of the GBA gene and ΨGBA pseudogene were amplified using PCR primers GDL444P-U6 with GDLseq-L2 or GDLseq-U4 with GDLseq-L1, respectively (see Table 1). PCR was completed using 2.5 U SuperTaq (Ambion), 1 ng template DNA, and 10 pmol each primer. Reactions consisted of 35 cycles of 94C for 30 s, 58 for 30 s, and 72C for 60 s followed by 2 minutes at 72C. PCR products were purified using a QIAquick PCR Purification Kit (Qiagen). Purified products were verified on Agilent Bioanalyzer for purity then were sent to ACTG, Inc. (Northbrook, IL) for dye-terminator DNA sequencing using each amplification primer. Sequences were aligned using the BCM Search Launcher web-based Multiple Sequence Alignment (ClustalW 1.8) application[24]. The alignment was formatted using Boxshade 3.21[25] with the following command line: "ulimit -t 30; box -def -numdef -out=wwwtmp/1.BOX.4270.9603.rtf -in=wwwtmp/1.BOX.4270.9603.ali -par = pt10.par -type = 1 -dev = 4 -thr = 0.8 >/dev/null".

## Results

With the goal of creating multiplexed techniques to offer reduced turnaround time and cost in multiple-disease assessment,[6] we developed a highly multiplexed suspension array to simultaneously identify 22 wild-type and 23 mutant alleles covering 8 diseases that are highly prevalent in the Ashkenazi Jewish population. This assay includes wild-type and mutant genetic determinations for Gaucher disease alleles c.1226A>G (N370S), 84GG, IVS2+1G>A, and c.1448T>C (L444P). In the assay, the c.1448T>C-containing region of the GBA gene was amplified by multiplex PCR using biotinylated primers. When amplifying GBA, it was crucial to distinguish gene from pseudogene. While the 55-bp deletion in ΨGBA located 502-nt upstream of the T to C transition might be used for assay systems amenable to large amplicons, [3-5] the length of amplicon created is not reported to be ideal for suspension arrays [8,9]. Hence, the design of new primers for amplifying the c.1448T>C region relied on reported sequence divergence between GBA and ΨGBA. The upstream primer was predicted to pair with both GBA and ΨGBA sequences. However, the downstream primer spanned nucleotide 7368 with its 3' terminus at nucleotide 7354 (reference sequence [GenBank:J03059.1]; [see additional file 1], Multiple sequence alignment of GBA and ΨGBA). The predicted 3' mispairing is expected to allow selective amplification of the gene and not the pseudogene [26], thus yielding a 134-bp amplicon based on GBA sequence. Amplified products were then directly hybridized to capture probes immobilized on fluorescently addressed microspheres [22]. After incubation with streptavidin-conjugated fluorophore, the reaction mixtures were analyzed by the Luminex flow cytometry platform to identify microsphere/amplicon interactions. Stringency was preserved by maintaining a reaction temperature of 52C. Allele ratios were calculated manually and by using Signature Script version 2.0 as described in the Materials and Methods section. Genetic determinations for c.1448T>C were assigned using the following parameters: allele ratios below 0.22 as normal, between 0.30 and 0.65 as heterozygous, above 0.75 as mutant, and intervening regions (0.22–0.30, 0.65–0.75) as indeterminate. The representative data shown in Table 2 suggest a robust methodology for identifying the allele of interest in DNA samples derived from cell line controls.

**Table 2:** Representative fluorescence and allele ratio developmental data

	*MFI*			
			
Cell Line Sample	c.1448T	c.1448T>C	Ratio	call
NA03461	1268	36	0.03	Normal
NA09960	1285	10	0.01	Normal
NA16193	1142	71	0.06	Normal
NA00852	1353	110	0.08	Normal
NA11215	1228	53	0.04	Normal^*a*^
NA10915	57	1804	0.97	c.1448T>C mut^*a*, *b*^

^*a*^Genotype sequence confirmed (data not shown)

^*b*^c.1448T>C homozygous mutant

Clinical evaluations were carried out to assess concordance with techniques already established in multiple reference laboratories. We expected to detect c.1448T>C at a frequency similar to that based on meta-analysis of the literature: between 1:340 and 1:1,200 (Table 3). At two independent clinical laboratories, Gaucher c.1448T>C allelic determinations were made for a total of 614 samples. Thirteen samples were observed to be heterozygous using our assay (see Table 4). All normal genotypes were concordant with independent results based on established, laboratory-validated methods reported by each respective laboratory (data not shown). However, all c.1448T>C heterozygous determinations (2.1%, 1:47) were discrepant with independent results reported by each respective laboratory. Hence, c.1448T>C heterozygous calls were observed about 7- to 25-fold more often than expected.

**Table 3:** Review of carrier frequencies of Gaucher allele c.1448T>C

		fraction		
			
carriers identified	patients assayed	decimal	1-in-	reference
0	1528	0.00	n/a	DeMarchi et al.[38]
4	1364	0.30	340	Eng et al.[39]
3	3764	0.08	1200	Kronn et al.[40]
0	572	0.00	n/a	Oddoux et al.[41]
1	546	0.20	550	Eng et al.[42]
3	3764	0.08	1200	Allitto et al.[43]
12	7706	0.10	640	Strom et al.[44]
17	17184	0.10	1010	Horowitz et al.[1]
				
40	36428	0.11	910	cumulative

**Table 4:** Representative fluorescence and allele ratio clinical data

	first design				second design			
	
	c.1448T	c.1448T>C	Ratio	call	c.1448T	c.1448T>C	Ratio	call
patient 4	1108	579	0.34	c.1448T>C het^*a*^	1249	54	0.04	Normal
patient 9	1068	585	0.35	c.1448T>C het	1091	93	0.08	Normal
patient 11	1273	679	0.35	c.1448T>C het	ND^*b*^	ND	ND	ND
patient 14	1229	654	0.35	c.1448T>C het	871	87	0.09	Normal
patient 17	1357	58	0.04	Normal	977	126	0.11	Normal
patient 27	1391	15	0.01	Normal	ND	ND	ND	ND
NA10915	57	1804	0.97	c.1448T>C mut^*c*^	5	1133	1.00	c.1448T>C mut
NA03461	1268	36	0.03	Normal	1228	0	0.00	Normal
NA08753	NA^*d*^	NA	NA	NA	844	603	0.42	c.1448T>C het

^*a*^c.1448T>C heterozygous

^*b*^Not Determined, limited sample volume exhausted during prior experimentation

^*c*^c.1448T>C homozygous mutant

^*d*^Not Available at the time of experimentation with first design

We suspected that the discrepancy was due to co-amplification of the functional gene and paralogous pseudogene due to a polymorphism in the primer-binding site of the pseudogene in discordant DNA samples. These predictions were borne out experimentally using DNA sequencing. A de-identified set of four discrepant c.1448T>C heterozygous samples and two concordant c.1448T normal samples were characterized further. A GBA gene-specific upstream primer was located within the paralogous 55 bp absent from exon 9 of ΨGBA. The ΨGBA pseudogene-specific upstream primer spanned the breakpoint of the 55-bp deletion. The regions of interest were amplified and sequenced for the GBA gene and ΨGBA pseudogene from each DNA sample along with a Coriell cell line DNA (accession number NA11215).

A multiple sequence alignment of the resultant sequences alongside those reported by Horowitz et al [1,4] and the UCSC Genome Bioinformatics database [16] is available as a supplemental file [see additional file 1]. The sequence data revealed several nucleotide positions that are not conserved between GBA and ΨGBA (Table 5). An analysis of each nucleotide position can be found in the discussion below.

**Table 5:** Nucleotide positions divergent between GBA and ΨGBA

		nucleotide position^*a*^							
		
gene	sequence	6844	7031	7159	7183	7192	7319	7354	7368
GBA	patient 4	G^*b*^	A/G	C	-	T	T	G	G
	patient 9	G	A/G	C	-	T	T	G	G
	patient 11	G	A/G	C	-	C/T	T	G	G
	patient 14	G	G	C	-	T	T	G	G
	patient 17	G	A/G	C	-	T	T	G	G
	patient 27	G	A/G	C	-	T	T	G	G
	NA11215	G	A/G	C	-	T	T	G	G
	Gen-4.87	G	G	C	G	T	T	G	G
	UCSC	G	A	C	-	T	T	G	G
									
ΨGBA	patient 4	C	G	C/T	-	C	C	G/C	G/C
	patient 9	C	G	T	-	C	C	G/C	G/C
	patient 11	C	G	C/T	-	C	C	G/C	G/C
	patient 14	C	G	C/T	-	C	C	G/C	G/C
	patient 17	C	G	T	-	C	C	C	C
	patient 27	C	G	T	-	C	C	C	C
	NA11215	C	A/G	T	-	C	C	C	C
	Gen-4.87	C	A	T	-	C	C	C	C
	UCSC	C	G	T	-	C	C	C	C

^*a*^Nucleotide positions are consistent with reference sequence [GenBank:J03059.1] (which is equivalent to that reported by the UCSC database for the region in question).[16] All nucleotides not listed displayed perfect identity between all nine sequences in the alignment.

^*b*^Nucleotide designations are G (guanosine), C (cytosine), T (thymidine), and A (adenosine). Observed heterozygous nucleotide positions are indicated as A/G, C/T, or G/C.

The nucleotide data observed at nucleotide positions 7354 and 7368 supported our hypothesis that a polymorphism in the primer-binding site of the pseudogene could allow co-amplification with the gene. As expected based on previous observations [1,15,16], all observed genes displayed a T at nucleotide 7319 and pseudogenes a C. Hence, these data further confirmed that if the pseudogene is co-amplified with the wild-type gene, both wild-type and mutant product will result. This set of products is then predicted to yield a heterozygous determination in the detection step.

As the only reliable distinguishing characteristic between gene and pseudogene, primers for the amplification of the region containing the c.1448T>C transition were positioned to span the upstream 55-bp deletion in the pseudogene. Amplification with these primers yielded a 576-bp amplicon, a length that could result in reduced hybridization and detection efficiencies in Luminex-based detection (S Dunbar, Luminex, personal communication; see also manufacturer recommendations [9]). However, the larger amplicon containing the c.1448T>C region yielded fluorescent signal of ~1000 in the hybridization assay as compared to ~1300 for the original product. Normal allele ratios for wild-type samples were also similar. Heterozygous detection for cell-line and patient samples was preserved. Heterozygosity due to amplification of the pseudogene was resolved for the original samples. These results are summarized in Table 4. Further testing of 280 new de-identified patient samples using this primer pair resulted in all normal c.1448T normal determinations (data not shown). Hence, successful detection of the larger amplicon appears to resolve issues with ΨGBA polymorphisms in this region.

## Discussion

The substantial growth of nucleic acid research in the last two decades has generated tremendous understanding of human disease. Advances in appreciation of the complex genetic factors involved in many diseases hold great promise for substantial improvements in medical diagnosis and treatment, but present challenges to the design of robust and reliable assays. For example, multifactorial illnesses have been characterized that are influenced by multiple genetic elements including genes and pseudogenes [18,27-31]. Study of the blood clotting disorder thrombophilia, for instance, requires characterization of numerous alleles within multiple genes that can be confounded by the presence of a pseudogene [32]. The complex nature of many such diseases has led to the development of multifaceted techniques that simultaneously examine many analytes. Multiplexing is the term used to describe the ability to analyze a number of analytes within a single process or assay. In addition to examining numerous components of a single disease, these same multiplex techniques have been adopted into research and clinical laboratories for assessing multiple inherited diseases within a given population. The benefits of consolidating multiple-disease testing into a single procedure such as the test presented in this work include reduced training, labor costs, and turnaround time [6].

In the design and evaluation of such reagents, care must be taken to minimize the occurrence of inaccurate detection. Accurate detection is desired both scientifically and ethically, since false positives can lead to unnecessary procedures, therapy, and psychosocial consequences [2,33,34].

In the interest of designing a multiplexed technique to offer reduced turnaround times and cost in the analysis of inherited diseases prevalent in the Ashkenazi population, we developed a set of reagents that allows genetic determinations for Gaucher disease alleles c.1226A>G (N370S), 84GG, IVS2+1G>A, and c.1448T>C (L444P). These reagents use multiplex PCR and bead array hybridization to facilitate an assay that detects 23 mutations per sample in fewer than 5 hours. Only a single transfer step is required with no intervening purification steps. Allele stringency is maintained without post-hybridization wash steps. In our preliminary design strategy, the c.1448T>C-targeting parallel primer matched both gene and pseudogene. The antiparallel primer was predicted to match the gene but not pair with the pseudogene at its 3' terminus, a strategy often used to selectively amplify particular sequences [26]. These predictions were borne out in all cell-line-based DNA controls assayed (Table 4 and data not shown). While such controls are helpful as preliminary confirmation of assay experimental design, they are not sufficient [35,36]. Cell line samples may not adequately represent the range of quality, quantity, and sequence identity of DNA used in a clinical setting. In contrast to single-source cell-lines, the genetic diversity of patient samples with potentially interfering polymorphisms may affect allele analysis.

Issues surrounding molecular analysis of the GBA gene in the invariable presence of ΨGBA have been reported [5]. For example, all pseudogene sequences published to date appear mutant at a site paralogous to the c.1448T>C mutation in the gene. In an effort to examine this and other polymorphisms and to obtain additional sequence for quality design, we sequenced GBA and ΨGBA from multiple DNA samples. As seen in Table 5, nucleotide 6844 (reference sequence [GenBank:J03059.1]) may be a candidate for reliable differentiation of gene from pseudogene during amplification; however, it offers little benefit over the 55-bp deletion which is tightly linked upstream and has been successfully used in techniques that allow larger amplicons [3-5]. Nucleotide 7031 is not reliable for the unique amplification of the gene. In fact, the UCSC database[15,16] and Horowitz et al[1] report discordant nucleotide identities at this site. This may be explained by our observation that some patients display both GBA and ΨGBA as heterozygous with A/G. Although all patients' genes exhibited C at nucleotide 7159, two patients' pseudogenes were heterozygous for C, rendering this an unreliable universal primer position for primer mispair-based differentiation. This nucleotide change – observed here in samples submitted for Ashkenazi carrier screening – has previously been reported in the Maya population [37]. The intronic "insertion" G (as compared to ΨGBA) reported by Horowitz et al[1,15,16] (nucleotide 7183) is neither reported in the UCSC database[15,16] nor observed in our sample set. Although all patients' pseudogenes exhibited C at nucleotide 7192, one patient's gene was heterozygous with C/T, rendering this another nucleotide position unreliable for differentiation. Nucleotide 7319 corresponds to the locus of interest (c.1448T). As expected based on previous reports,[1,15-17] all sequenced genes had T and all pseudogenes had C at this site. No downstream nucleotide positions were discovered during this analysis that could be reliably used for GBA/ΨGBA mispair-based differentiation during amplification.

The data observed at nucleotides 7354 and 7368 supported our hypothesis that a polymorphism in the primer-binding site of the pseudogene could allow co-amplification with the gene. Four patient samples displayed G in the gene at these sites and G/C in the pseudogene. Hence, the four samples that yielded discrepant c.1448T>C heterozygous determinations have sequences in their pseudogenes that are predicted to be amplified by our original primer design. Since pseudogenes from these same patients were sequence confirmed to contain C at nucleotide 7319, their amplified products are predicted to bind to c.1448T>C mutant capture probes. This would then be expected to result in the heterozygous determinations seen in these patients. Only by using a well established region of differentiation between GBA and ΨGBA were we able to reliably enrich for gene-specific amplification. While this approach created a larger amplified product, it was sufficient for detection on a suspension bead array platform.

## Conclusion

Our experience demonstrates the limitations of using publicly-available sequence databases for multiplex assay design. We conclude that for gene/pseudogene differentiation it may be insufficient to use the sequence information currently available without additional clinical testing. As multiple, unique genomic DNA sequence information is not always readily available, the sequence data contributed here should aid in the future design of genetic assays in this region of GBA. Furthermore, whereas the available sequence information suggested discrimination capabilities with a shorter product, we identified a single-nucleotide polymorphism in the pseudogene that required the use of a larger product for effective gene/pseudogene discrimination. We successfully demonstrated that larger amplified products can be directly hybridized to bead-bound capture probes in a liquid bead array. With regards to the complexity of genetic testing and the increased use of multiplexed platforms, this report urges caution when designing tests for Gaucher disease and other genetic assays dependent on the frequency of SNPs that may not be readily apparent based on current genomic sequence database releases. This report also underscores the continued need for thorough clinical evaluation of new assays prior to adoption into routine testing. We advocate multiple-laboratory clinical evaluation of assay designs early in the development process to produce robust and reliable multiplex diagnostic assays.

## Competing interests

The authors have received salary from their declared affiliation – Asuragen – an organization that may benefit from the publication of this manuscript. Asuragen is funding all costs for this manuscript.

## Authors' contributions

JB acted as project leader, drafted the manuscript and performed experimental design and analysis. CL performed bead array benchwork and analysis. WL-W performed the sequencing effort. AG consulted on project design and contributed substantially to the drafting of the manuscript. All authors read and approved the final manuscript.

## Pre-publication history

The pre-publication history for this paper can be accessed here:



## Supplementary Material

Additional File 1 Multiple sequence alignment of GBA and ΨGBA. The c.1448T>C (L444P) regions of the GBA gene and ΨGBA pseudogene were amplified by PCR then sequenced. Sequences were aligned using the BCM Search Launcher web-based Multiple Sequence Alignment (ClustalW 1.8) application [24]. The alignment was formatted using Boxshade 3.21 [25]. Nucleotide positions of divergent nucleotides are consistent with reference sequence [GenBank:J03059.1] (which is equivalent to that reported by the UCSC database for the region in question) [16]. Nucleotides shown in white text on black background displayed identity between at least 80% of the 18 sequences in the alignment. Nucleotide designations are G (guanosine), C (cytosine), T (thymidine), and A (adenosine). Heterozygous nucleotide positions are indicated as R (A/G), Y (C/T), or S (G/C). Click here for file
