# Supplementary material for: Polymorphisms in the glucocerebrosidase gene and pseudogene urge caution in clinical analysis of Gaucher disease allele c.1448T>C (L444P)
Source: BMC Med Genet. 2006 Aug 3;7:69. doi: 10.1186/1471-2350-7-69 (PMC1559599; doi:10.1186/1471-2350-7-69)
Supplement: Additional File 1 — Multiple sequence alignment of GBA and ΨGBA. The c.1448T>C (L444P) regions of the GBA gene and ΨGBA pseudogene were amplified by PCR then sequenced. Sequences were aligned using the BCM Search Launcher web-based Multiple Sequence Alignment (ClustalW 1.8) application [24]. The alignment was formatted using Boxshade 3.21 [25]. Nucleotide positions of divergent nucleotides are consistent with reference sequence [GenBank:J03059.1] (which is equivalent to that reported by the UCSC database for the region in question) [16]. Nucleotides shown in white text on black background displayed identity between at least 80% of the 18 sequences in the alignment. Nucleotide designations are G (guanosine), C (cytosine), T (thymidine), and A (adenosine). Heterozygous nucleotide positions are indicated as R (A/G), Y (C/T), or S (G/C). [file 1471-2350-7-69-S1.pdf]

**Multiple sequence alignment of GBA and ΨGBA**

The L444P regions of the GBA gene and ΨGBA pseudogene were amplified by PCR then sequenced. Sequences were aligned using the BCM Search Launcher web-based Multiple Sequence Alignment (ClustalW 1.8) application [1]. The alignment was formatted using Boxshade 3.21 [2]. Nucleotide positions of divergent nucleotides are consistent with NCBI accession number J03059 (which is equivalent to that reported by the UCSC database for the region in question) [3]. Nucleotides shown in white text on black background displayed identity between at least 80% of the 18 sequences in the alignment. Nucleotide designations are G (guanosine), C (cytosine), T (thymidine), and A (adenosine). Heterozygous nucleotide positions are indicated as R (A/G), Y (C/T), or S (G/C).

**Supplemental file references**


1. Smith RF, Wiese BA, Wojzynski MK, Davison DB, Worley KC: **BCM Search Launcher--an integrated interface to molecular biology data base search and analysis services available on the World Wide Web**. *Genome Res* 1996, **6**(5):454-462.
2. **BOXSHADE 3.21 Pretty Printing and Shading of Multiple-Alignment files** [http://www.ch.embnet.org/software/BOX_form.html]
3. Karolchik D, Baertsch R, Diekhans M, Furey TS, Hinrichs A, Lu YT, Roskin KM, Schwartz M, Sugnet CW, Thomas DJ *et al*: **The UCSC Genome Browser Database**. *Nucleic Acids Res* 2003, **31**(1):51-54.

Supplemental file

```
                                   6844
Sample4g      1 CATTGTAGACATCACCAAGGACACGTTTTACAAACAGCCCATGTTCTACCACCTTGGCCA
Sample9g      1 CATTGTAGACATCACCAAGGACACGTTTTACAAACAGCCCATGTTCTACCACCTTGGCCA
Sample11g     1 CATTGTAGACATCACCAAGGACACGTTTTACAAACAGCCCATGTTCTACCACCTTGGCCA
Sample14g     1 CATTGTAGACATCACCAAGGACACGTTTTACAAACAGCCCATGTTCTACCACCTTGGCCA
Sample17g     1 CATTGTAGACATCACCAAGGACACGTTTTACAAACAGCCCATGTTCTACCACCTTGGCCA
Sample27g     1 CATTGTAGACATCACCAAGGACACGTTTTACAAACAGCCCATGTTCTACCACCTTGGCCA
NA11215g      1 CATTGTAGACATCACCAAGGACACGTTTTACAAACAGCCCATGTTCTACCACCTTGGCCA
Gen-4.87g     1 CATTGTAGACATCACCAAGGACACGTTTTACAAACAGCCCATGTTCTACCACCTTGGCCA
UCSCg         1 CATTGTAGACATCACCAAGGACACGTTTTACAAACAGCCCATGTTCTACCACCTTGGCCA
Sample4p      1 CATTGTAGACATCACCAAGCACACGTTTTACAAACAGCCCATGTTCTACCACCTTGGCCA
Sample9p      1 CATTGTAGACATCACCAAGCACACGTTTTACAAACAGCCCATGTTCTACCACCTTGGCCA
Sample11p     1 CATTGTAGACATCACCAAGCACACGTTTTACAAACAGCCCATGTTCTACCACCTTGGCCA
Sample14p     1 CATTGTAGACATCACCAAGCACACGTTTTACAAACAGCCCATGTTCTACCACCTTGGCCA
Sample17p     1 CATTGTAGACATCACCAAGCACACGTTTTACAAACAGCCCATGTTCTACCACCTTGGCCA
Sample27p     1 CATTGTAGACATCACCAAGCACACGTTTTACAAACAGCCCATGTTCTACCACCTTGGCCA
NA11215p      1 CATTGTAGACATCACCAAGCACACGTTTTACAAACAGCCCATGTTCTACCACCTTGGCCA
Gen-4.87p     1 CATTGTAGACATCACCAAGCACACGTTTTACAAACAGCCCATGTTCTACCACCTTGGCCA
UCSCp         1 CATTGTAGACATCACCAAGCACACGTTTTACAAACAGCCCATGTTCTACCACCTTGGCCA

Sample4g     61 CTTCAGGTGAGTGGAGGGCGGGCACCCCCATTCCATACCAGGCCTATCATCTCCTACATC
Sample9g     61 CTTCAGGTGAGTGGAGGGCGGGCACCCCCATTCCATACCAGGCCTATCATCTCCTACATC
Sample11g    61 CTTCAGGTGAGTGGAGGGCGGGCACCCCCATTCCATACCAGGCCTATCATCTCCTACATC
Sample14g    61 CTTCAGGTGAGTGGAGGGCGGGCACCCCCATTCCATACCAGGCCTATCATCTCCTACATC
Sample17g    61 CTTCAGGTGAGTGGAGGGCGGGCACCCCCATTCCATACCAGGCCTATCATCTCCTACATC
Sample27g    61 CTTCAGGTGAGTGGAGGGCGGGCACCCCCATTCCATACCAGGCCTATCATCTCCTACATC
NA11215g     61 CTTCAGGTGAGTGGAGGGCGGGCACCCCCATTCCATACCAGGCCTATCATCTCCTACATC
Gen-4.87g    61 CTTCAGGTGAGTGGAGGGCGGGCACCCCCATTCCATACCAGGCCTATCATCTCCTACATC
UCSCg        61 CTTCAGGTGAGTGGAGGGCGGGCACCCCCATTCCATACCAGGCCTATCATCTCCTACATC
Sample4p     61 CTTCAGGTGAGTGGAGGGCGGGCACCCCCATTCCATACCAGGCCTATCATCTCCTACATC
Sample9p     61 CTTCAGGTGAGTGGAGGGCGGGCACCCCCATTCCATACCAGGCCTATCATCTCCTACATC
Sample11p    61 CTTCAGGTGAGTGGAGGGCGGGCACCCCCATTCCATACCAGGCCTATCATCTCCTACATC
Sample14p    61 CTTCAGGTGAGTGGAGGGCGGGCACCCCCATTCCATACCAGGCCTATCATCTCCTACATC
Sample17p    61 CTTCAGGTGAGTGGAGGGCGGGCACCCCCATTCCATACCAGGCCTATCATCTCCTACATC
Sample27p    61 CTTCAGGTGAGTGGAGGGCGGGCACCCCCATTCCATACCAGGCCTATCATCTCCTACATC
NA11215p     61 CTTCAGGTGAGTGGAGGGCGGGCACCCCCATTCCATACCAGGCCTATCATCTCCTACATC
Gen-4.87p    61 CTTCAGGTGAGTGGAGGGCGGGCACCCCCATTCCATACCAGGCCTATCATCTCCTACATC
UCSCp        61 CTTCAGGTGAGTGGAGGGCGGGCACCCCCATTCCATACCAGGCCTATCATCTCCTACATC

Sample4g    121 GGATGGCTTACATCACTCTACACCACGAGGGAGCAGGAAGGTGTTCAGGGTGGAACCTCG
Sample9g    121 GGATGGCTTACATCACTCTACACCACGAGGGAGCAGGAAGGTGTTCAGGGTGGAACCTCG
Sample11g   121 GGATGGCTTACATCACTCTACACCACGAGGGAGCAGGAAGGTGTTCAGGGTGGAACCTCG
Sample14g   121 GGATGGCTTACATCACTCTACACCACGAGGGAGCAGGAAGGTGTTCAGGGTGGAACCTCG
Sample17g   121 GGATGGCTTACATCACTCTACACCACGAGGGAGCAGGAAGGTGTTCAGGGTGGAACCTCG
Sample27g   121 GGATGGCTTACATCACTCTACACCACGAGGGAGCAGGAAGGTGTTCAGGGTGGAACCTCG
NA11215g    121 GGATGGCTTACATCACTCTACACCACGAGGGAGCAGGAAGGTGTTCAGGGTGGAACCTCG
Gen-4.87g   121 GGATGGCTTACATCACTCTACACCACGAGGGAGCAGGAAGGTGTTCAGGGTGGAACCTCG
UCSCg       121 GGATGGCTTACATCACTCTACACCACGAGGGAGCAGGAAGGTGTTCAGGGTGGAACCTCG
Sample4p    121 GGATGGCTTACATCACTCTACACCACGAGGGAGCAGGAAGGTGTTCAGGGTGGAACCTCG
Sample9p    121 GGATGGCTTACATCACTCTACACCACGAGGGAGCAGGAAGGTGTTCAGGGTGGAACCTCG
Sample11p   121 GGATGGCTTACATCACTCTACACCACGAGGGAGCAGGAAGGTGTTCAGGGTGGAACCTCG
Sample14p   121 GGATGGCTTACATCACTCTACACCACGAGGGAGCAGGAAGGTGTTCAGGGTGGAACCTCG
Sample17p   121 GGATGGCTTACATCACTCTACACCACGAGGGAGCAGGAAGGTGTTCAGGGTGGAACCTCG
Sample27p   121 GGATGGCTTACATCACTCTACACCACGAGGGAGCAGGAAGGTGTTCAGGGTGGAACCTCG
NA11215p    121 GGATGGCTTACATCACTCTACACCACGAGGGAGCAGGAAGGTGTTCAGGGTGGAACCTCG
Gen-4.87p   121 GGATGGCTTACATCACTCTACACCACGAGGGAGCAGGAAGGTGTTCAGGGTGGAACCTCG
UCSCp       121 GGATGGCTTACATCACTCTACACCACGAGGGAGCAGGAAGGTGTTCAGGGTGGAACCTCG
```

Supplemental file (continued)

```
                                           7031
Sample4g    181 GAAGAGGCACACCCATCCCCTTTTGCRCCATGGAGGCAGGAAGTGACTAGGTAGCAACAG
Sample9g    181 GAAGAGGCACACCCATCCCCTTTTGCRCCATGGAGGCAGGAAGTGACTAGGTAGCAACAG
Sample11g   181 GAAGAGGCACACCCATCCCCTTTTGCRCCATGGAGGCAGGAAGTGACTAGGTAGCAACAG
Sample14g   181 GAAGAGGCACACCCATCCCCTTTTGCGCCATGGAGGCAGGAAGTGACTAGGTAGCAACAG
Sample17g   181 GAAGAGGCACACCCATCCCCTTTTGCRCCATGGAGGCAGGAAGTGACTAGGTAGCAACAG
Sample27g   181 GAAGAGGCACACCCATCCCCTTTTGCRCCATGGAGGCAGGAAGTGACTAGGTAGCAACAG
NA11215g    181 GAAGAGGCACACCCATCCCCTTTTGCRCCATGGAGGCAGGAAGTGACTAGGTAGCAACAG
Gen-4.87g   181 GAAGAGGCACACCCATCCCCTTTTGCGCCATGGAGGCAGGAAGTGACTAGGTAGCAACAG
UCSCg       181 GAAGAGGCACACCCATCCCCTTTTGCACCATGGAGGCAGGAAGTGACTAGGTAGCAACAG
Sample4p    181 GAAGAGGCACACCCATCCCCTTTTGCGCCATGGAGGCAGGAAGTGACTAGGTAGCAACAG
Sample9p    181 GAAGAGGCACACCCATCCCCTTTTGCGCCATGGAGGCAGGAAGTGACTAGGTAGCAACAG
Sample11p   181 GAAGAGGCACACCCATCCCCTTTTGCGCCATGGAGGCAGGAAGTGACTAGGTAGCAACAG
Sample14p   181 GAAGAGGCACACCCATCCCCTTTTGCGCCATGGAGGCAGGAAGTGACTAGGTAGCAACAG
Sample17p   181 GAAGAGGCACACCCATCCCCTTTTGCGCCATGGAGGCAGGAAGTGACTAGGTAGCAACAG
Sample27p   181 GAAGAGGCACACCCATCCCCTTTTGCGCCATGGAGGCAGGAAGTGACTAGGTAGCAACAG
NA11215p    181 GAAGAGGCACACCCATCCCCTTTTGCRCCATGGAGGCAGGAAGTGACTAGGTAGCAACAG
Gen-4.87p   181 GAAGAGGCACACCCATCCCCTTTTGCACCATGGAGGCAGGAAGTGACTAGGTAGCAACAG
UCSCp       181 GAAGAGGCACACCCATCCCCTTTTGCGCCATGGAGGCAGGAAGTGACTAGGTAGCAACAG

Sample4g    241 AAAACCCCAATGCCTGAGGCTGGACTGCGATGCAGAAAAGCAGGGTCAGTGCCCAGCAGC
Sample9g    241 AAAACCCCAATGCCTGAGGCTGGACTGCGATGCAGAAAAGCAGGGTCAGTGCCCAGCAGC
Sample11g   241 AAAACCCCAATGCCTGAGGCTGGACTGCGATGCAGAAAAGCAGGGTCAGTGCCCAGCAGC
Sample14g   241 AAAACCCCAATGCCTGAGGCTGGACTGCGATGCAGAAAAGCAGGGTCAGTGCCCAGCAGC
Sample17g   241 AAAACCCCAATGCCTGAGGCTGGACTGCGATGCAGAAAAGCAGGGTCAGTGCCCAGCAGC
Sample27g   241 AAAACCCCAATGCCTGAGGCTGGACTGCGATGCAGAAAAGCAGGGTCAGTGCCCAGCAGC
NA11215g    241 AAAACCCCAATGCCTGAGGCTGGACTGCGATGCAGAAAAGCAGGGTCAGTGCCCAGCAGC
Gen-4.87g   241 AAAACCCCAATGCCTGAGGCTGGACTGCGATGCAGAAAAGCAGGGTCAGTGCCCAGCAGC
UCSCg       241 AAAACCCCAATGCCTGAGGCTGGACTGCGATGCAGAAAAGCAGGGTCAGTGCCCAGCAGC
Sample4p    241 AAAACCCCAATGCCTGAGGCTGGACTGCGATGCAGAAAAGCAGGGTCAGTGCCCAGCAGC
Sample9p    241 AAAACCCCAATGCCTGAGGCTGGACTGCGATGCAGAAAAGCAGGGTCAGTGCCCAGCAGC
Sample11p   241 AAAACCCCAATGCCTGAGGCTGGACTGCGATGCAGAAAAGCAGGGTCAGTGCCCAGCAGC
Sample14p   241 AAAACCCCAATGCCTGAGGCTGGACTGCGATGCAGAAAAGCAGGGTCAGTGCCCAGCAGC
Sample17p   241 AAAACCCCAATGCCTGAGGCTGGACTGCGATGCAGAAAAGCAGGGTCAGTGCCCAGCAGC
Sample27p   241 AAAACCCCAATGCCTGAGGCTGGACTGCGATGCAGAAAAGCAGGGTCAGTGCCCAGCAGC
NA11215p    241 AAAACCCCAATGCCTGAGGCTGGACTGCGATGCAGAAAAGCAGGGTCAGTGCCCAGCAGC
Gen-4.87p   241 AAAACCCCAATGCCTGAGGCTGGACTGCGATGCAGAAAAGCAGGGTCAGTGCCCAGCAGC
UCSCp       241 AAAACCCCAATGCCTGAGGCTGGACTGCGATGCAGAAAAGCAGGGTCAGTGCCCAGCAGC

                                                   7159                    7183
Sample4g    301 ATGGCTCCAGGCCTAGAGAGCCAGGGCAGAGCCTCTGCAGGAGTTATGGGGTGGGTCCG-
Sample9g    301 ATGGCTCCAGGCCTAGAGAGCCAGGGCAGAGCCTCTGCAGGAGTTATGGGGTGGGTCCG-
Sample11g   301 ATGGCTCCAGGCCTAGAGAGCCAGGGCAGAGCCTCTGCAGGAGTTATGGGGTGGGTCCG-
Sample14g   301 ATGGCTCCAGGCCTAGAGAGCCAGGGCAGAGCCTCTGCAGGAGTTATGGGGTGGGTCCG-
Sample17g   301 ATGGCTCCAGGCCTAGAGAGCCAGGGCAGAGCCTCTGCAGGAGTTATGGGGTGGGTCCG-
Sample27g   301 ATGGCTCCAGGCCTAGAGAGCCAGGGCAGAGCCTCTGCAGGAGTTATGGGGTGGGTCCG-
NA11215g    301 ATGGCTCCAGGCCTAGAGAGCCAGGGCAGAGCCTCTGCAGGAGTTATGGGGTGGGTCCG-
Gen-4.87g   301 ATGGCTCCAGGCCTAGAGAGCCAGGGCAGAGCCTCTGCAGGAGTTATGGGGTGGGTCCGG
UCSCg       301 ATGGCTCCAGGCCTAGAGAGCCAGGGCAGAGCCTCTGCAGGAGTTATGGGGTGGGTCCG-
Sample4p    301 ATGGCTCCAGGCCTAGAGAGCCAGGGCAGAGCCTYTGCAGGAGTTATGGGGTGGGTCCG-
Sample9p    301 ATGGCTCCAGGCCTAGAGAGCCAGGGCAGAGCCTTTGCAGGAGTTATGGGGTGGGTCCG-
Sample11p   301 ATGGCTCCAGGCCTAGAGAGCCAGGGCAGAGCCTYTGCAGGAGTTATGGGGTGGGTCCG-
Sample14p   301 ATGGCTCCAGGCCTAGAGAGCCAGGGCAGAGCCTYTGCAGGAGTTATGGGGTGGGTCCG-
Sample17p   301 ATGGCTCCAGGCCTAGAGAGCCAGGGCAGAGCCTTTGCAGGAGTTATGGGGTGGGTCCG-
Sample27p   301 ATGGCTCCAGGCCTAGAGAGCCAGGGCAGAGCCTTTGCAGGAGTTATGGGGTGGGTCCG-
NA11215p    301 ATGGCTCCAGGCCTAGAGAGCCAGGGCAGAGCCTTTGCAGGAGTTATGGGGTGGGTCCG-
Gen-4.87p   301 ATGGCTCCAGGCCTAGAGAGCCAGGGCAGAGCCTTTGCAGGAGTTATGGGGTGGGTCCG-
UCSCp       301 ATGGCTCCAGGCCTAGAGAGCCAGGGCAGAGCCTTTGCAGGAGTTATGGGGTGGGTCCG-
```

Supplemental file (continued)

```
                     7192
Sample4g    360 TGGGTGGGTGACTTCTTAGATGAGGGTTTCATGGGAGGTACCCCGAGGGACTCTGACCAT
Sample9g    360 TGGGTGGGTGACTTCTTAGATGAGGGTTTCATGGGAGGTACCCCGAGGGACTCTGACCAT
Sample11g   360 TGGGTGGGYGACTTCTTAGATGAGGGTTTCATGGGAGGTACCCCGAGGGACTCTGACCAT
Sample14g   360 TGGGTGGGTGACTTCTTAGATGAGGGTTTCATGGGAGGTACCCCGAGGGACTCTGACCAT
Sample17g   360 TGGGTGGGTGACTTCTTAGATGAGGGTTTCATGGGAGGTACCCCGAGGGACTCTGACCAT
Sample27g   360 TGGGTGGGTGACTTCTTAGATGAGGGTTTCATGGGAGGTACCCCGAGGGACTCTGACCAT
NA11215g    360 TGGGTGGGTGACTTCTTAGATGAGGGTTTCATGGGAGGTACCCCGAGGGACTCTGACCAT
Gen-4.87g   361 TGGGTGGGTGACTTCTTAGATGAGGGTTTCATGGGAGGTACCCCGAGGGACTCTGACCAT
UCSCg       360 TGGGTGGGTGACTTCTTAGATGAGGGTTTCATGGGAGGTACCCCGAGGGACTCTGACCAT
Sample4p    360 TGGGTGGGCGACTTCTTAGATGAGGGTTTCATGGGAGGTACCCCGAGGGACTCTGACCAT
Sample9p    360 TGGGTGGGCGACTTCTTAGATGAGGGTTTCATGGGAGGTACCCCGAGGGACTCTGACCAT
Sample11p   360 TGGGTGGGCGACTTCTTAGATGAGGGTTTCATGGGAGGTACCCCGAGGGACTCTGACCAT
Sample14p   360 TGGGTGGGCGACTTCTTAGATGAGGGTTTCATGGGAGGTACCCCGAGGGACTCTGACCAT
Sample17p   360 TGGGTGGGCGACTTCTTAGATGAGGGTTTCATGGGAGGTACCCCGAGGGACTCTGACCAT
Sample27p   360 TGGGTGGGCGACTTCTTAGATGAGGGTTTCATGGGAGGTACCCCGAGGGACTCTGACCAT
NA11215p    360 TGGGTGGGCGACTTCTTAGATGAGGGTTTCATGGGAGGTACCCCGAGGGACTCTGACCAT
Gen-4.87p   360 TGGGTGGGCGACTTCTTAGATGAGGGTTTCATGGGAGGTACCCCGAGGGACTCTGACCAT
UCSCp       360 TGGGTGGGCGACTTCTTAGATGAGGGTTTCATGGGAGGTACCCCGAGGGACTCTGACCAT

Sample4g    420 CTGTTCCCACATTCAGCAAGTTCATTCCTGAGGGCTCCCAGAGAGTGGGGCTGGTTGCCA
Sample9g    420 CTGTTCCCACATTCAGCAAGTTCATTCCTGAGGGCTCCCAGAGAGTGGGGCTGGTTGCCA
Sample11g   420 CTGTTCCCACATTCAGCAAGTTCATTCCTGAGGGCTCCCAGAGAGTGGGGCTGGTTGCCA
Sample14g   420 CTGTTCCCACATTCAGCAAGTTCATTCCTGAGGGCTCCCAGAGAGTGGGGCTGGTTGCCA
Sample17g   420 CTGTTCCCACATTCAGCAAGTTCATTCCTGAGGGCTCCCAGAGAGTGGGGCTGGTTGCCA
Sample27g   420 CTGTTCCCACATTCAGCAAGTTCATTCCTGAGGGCTCCCAGAGAGTGGGGCTGGTTGCCA
NA11215g    420 CTGTTCCCACATTCAGCAAGTTCATTCCTGAGGGCTCCCAGAGAGTGGGGCTGGTTGCCA
Gen-4.87g   421 CTGTTCCCACATTCAGCAAGTTCATTCCTGAGGGCTCCCAGAGAGTGGGGCTGGTTGCCA
UCSCg       420 CTGTTCCCACATTCAGCAAGTTCATTCCTGAGGGCTCCCAGAGAGTGGGGCTGGTTGCCA
Sample4p    420 CTGTTCCCACATTCAGCAAGTTCATTCCTGAGGGCTCCCAGAGAGTGGGGCTGGTTGCCA
Sample9p    420 CTGTTCCCACATTCAGCAAGTTCATTCCTGAGGGCTCCCAGAGAGTGGGGCTGGTTGCCA
Sample11p   420 CTGTTCCCACATTCAGCAAGTTCATTCCTGAGGGCTCCCAGAGAGTGGGGCTGGTTGCCA
Sample14p   420 CTGTTCCCACATTCAGCAAGTTCATTCCTGAGGGCTCCCAGAGAGTGGGGCTGGTTGCCA
Sample17p   420 CTGTTCCCACATTCAGCAAGTTCATTCCTGAGGGCTCCCAGAGAGTGGGGCTGGTTGCCA
Sample27p   420 CTGTTCCCACATTCAGCAAGTTCATTCCTGAGGGCTCCCAGAGAGTGGGGCTGGTTGCCA
NA11215p    420 CTGTTCCCACATTCAGCAAGTTCATTCCTGAGGGCTCCCAGAGAGTGGGGCTGGTTGCCA
Gen-4.87p   420 CTGTTCCCACATTCAGCAAGTTCATTCCTGAGGGCTCCCAGAGAGTGGGGCTGGTTGCCA
UCSCp       420 CTGTTCCCACATTCAGCAAGTTCATTCCTGAGGGCTCCCAGAGAGTGGGGCTGGTTGCCA

                            7319                               7354
Sample4g    480 GTCAGAAGAACGACCTGGACGCAGTGGCACTGATGCATCCCGATGGCTCTGCTGTTGTGG
Sample9g    480 GTCAGAAGAACGACCTGGACGCAGTGGCACTGATGCATCCCGATGGCTCTGCTGTTGTGG
Sample11g   480 GTCAGAAGAACGACCTGGACGCAGTGGCACTGATGCATCCCGATGGCTCTGCTGTTGTGG
Sample14g   480 GTCAGAAGAACGACCTGGACGCAGTGGCACTGATGCATCCCGATGGCTCTGCTGTTGTGG
Sample17g   480 GTCAGAAGAACGACCTGGACGCAGTGGCACTGATGCATCCCGATGGCTCTGCTGTTGTGG
Sample27g   480 GTCAGAAGAACGACCTGGACGCAGTGGCACTGATGCATCCCGATGGCTCTGCTGTTGTGG
NA11215g    480 GTCAGAAGAACGACCTGGACGCAGTGGCACTGATGCATCCCGATGGCTCTGCTGTTGTGG
Gen-4.87g   481 GTCAGAAGAACGACCTGGACGCAGTGGCACTGATGCATCCCGATGGCTCTGCTGTTGTGG
UCSCg       480 GTCAGAAGAACGACCTGGACGCAGTGGCACTGATGCATCCCGATGGCTCTGCTGTTGTGG
Sample4p    480 GTCAGAAGAACGACCCGGACGCAGTGGCACTGATGCATCCCGATGGCTCTSCTGTTGTGG
Sample9p    480 GTCAGAAGAACGACCCGGACGCAGTGGCACTGATGCATCCCGATGGCTCTSCTGTTGTGG
Sample11p   480 GTCAGAAGAACGACCCGGACGCAGTGGCACTGATGCATCCCGATGGCTCTSCTGTTGTGG
Sample14p   480 GTCAGAAGAACGACCCGGACGCAGTGGCACTGATGCATCCCGATGGCTCTSCTGTTGTGG
Sample17p   480 GTCAGAAGAACGACCCGGACGCAGTGGCACTGATGCATCCCGATGGCTCTCCTGTTGTGG
Sample27p   480 GTCAGAAGAACGACCCGGACGCAGTGGCACTGATGCATCCCGATGGCTCTCCTGTTGTGG
NA11215p    480 GTCAGAAGAACGACCCGGACGCAGTGGCACTGATGCATCCCGATGGCTCTCCTGTTGTGG
Gen-4.87p   480 GTCAGAAGAACGACCCGGACGCAGTGGCACTGATGCATCCCGATGGCTCTCCTGTTGTGG
UCSCp       480 GTCAGAAGAACGACCCGGACGCAGTGGCACTGATGCATCCCGATGGCTCTCCTGTTGTGG
```

Supplemental file (continued)

```
                    7368
Sample4g    540 TCGTGCTAAACCGGTGAGGGCAATGGTGAGGTCTGGGAAGTGGGCTGAAGACAGCGTTGG
Sample9g    540 TCGTGCTAAACCGGTGAGGGCAATGGTGAGGTCTGGGAAGTGGGCTGAAGACAGCGTTGG
Sample11g   540 TCGTGCTAAACCGGTGAGGGCAATGGTGAGGTCTGGGAAGTGGGCTGAAGACAGCGTTGG
Sample14g   540 TCGTGCTAAACCGGTGAGGGCAATGGTGAGGTCTGGGAAGTGGGCTGAAGACAGCGTTGG
Sample17g   540 TCGTGCTAAACCGGTGAGGGCAATGGTGAGGTCTGGGAAGTGGGCTGAAGACAGCGTTGG
Sample27g   540 TCGTGCTAAACCGGTGAGGGCAATGGTGAGGTCTGGGAAGTGGGCTGAAGACAGCGTTGG
NA11215g    540 TCGTGCTAAACCGGTGAGGGCAATGGTGAGGTCTGGGAAGTGGGCTGAAGACAGCGTTGG
Gen-4.87g   541 TCGTGCTAAACCGGTGAGGGCAATGGTGAGGTCTGGGAAGTGGGCTGAAGACAGCGTTGG
UCSCg       540 TCGTGCTAAACCGGTGAGGGCAATGGTGAGGTCTGGGAAGTGGGCTGAAGACAGCGTTGG
Sample4p    540 TCGTSCTAAACCGGTGAGGGCAATGGTGAGGTCTGGGAAGTGGGCTGAAGACAGCGTTGG
Sample9p    540 TCGTSCTAAACCGGTGAGGGCAATGGTGAGGTCTGGGAAGTGGGCTGAAGACAGCGTTGG
Sample11p   540 TCGTSCTAAACCGGTGAGGGCAATGGTGAGGTCTGGGAAGTGGGCTGAAGACAGCGTTGG
Sample14p   540 TCGTSCTAAACCGGTGAGGGCAATGGTGAGGTCTGGGAAGTGGGCTGAAGACAGCGTTGG
Sample17p   540 TCGTCCTAAACCGGTGAGGGCAATGGTGAGGTCTGGGAAGTGGGCTGAAGACAGCGT---
Sample27p   540 TCGTCCTAAACCGGTGAGGGCAATGGTGAGGTCTGGGAAGTGGGCTGAAGACAGCGTTGG
NA11215p    540 TCGTCCTAAACCGGTGAGGGCAATGGTGAGGTCTGGGAAGTGGGCTGAAGACAGCGTTGG
Gen-4.87p   540 TCGTCCTAAACCGGTGAGGGCAATGGTGAGGTCTGGGAAGTGGGCTGAAGACAGCGTTGG
UCSCp       540 TCGTCCTAAACCGGTGAGGGCAATGGTGAGGTCTGGGAAGTGGGCTGAAGACAGCGTTGG


Sample4g    600 GGGCCTTGGCAGGATCAC
Sample9g    600 GGGCCTTGGCAGGATCAC
Sample11g   600 GGGCCTTGGCAGGATCAC
Sample14g   600 GGGCCTTGGCAGGATCAC
Sample17g   600 GGGCCTTGGCAGGATCAC
Sample27g   600 GGGCCTTGGCAGGATCAC
NA11215g    600 GGGCCTTGGCAGGATCAC
Gen-4.87g   601 GGGCCTTGGCAGGATCAC
UCSCg       600 GGGCCTTGGCAGGATCAC
Sample4p    600 GGGCCTTGGCAGGATCAC
Sample9p    600 GGGCCTTGGCAGGATCAC
Sample11p   600 GGGCCTTGGCAGGATCAC
Sample14p   600 GGGCCTTGGCAGGATCA-
Sample17p       ------------------
Sample27p   600 GGGCCTTGGCAGGATCAC
NA11215p    600 GGGCCTTGGCAGGAT---
Gen-4.87p   600 GGGCCTTGGCAGGATCAC
UCSCp       600 GGGCCTTGGCAGGATCAC
```
